# Development of a nomogram for predicting axillary pathologic complete response after neoadjuvant therapy in clinically node-positive breast cancer

**DOI:** 10.3389/fonc.2026.1937807

**Published:** 2026-08-21

**Authors:** Bin Li, An Yi, Tenghua Yu, Yongqiang Bao, Lihong He, Zhuoyu He, Jin Liu, Yan Liu, Chunling Jiang

**Affiliations:** 1 Jiangxi Medical College, Nanchang University, Nanchang, China; 2 Department of Radiation Oncology Jiangxi Cancer Hospital & Institute, Jiangxi Clinical Research Center for Cancer, The Second Affiliated Hospital of Nanchang Medical College;, Nanchang, China; 3 Yichun Vocational Technical College of Medicine, The Affiliated Hospital of Yichun Vocational Technical College, Yichun, China; 4 Department of Breast Surgery, Jiangxi Cancer Hospital & Institute, The Second Affiliated Hospital of Nanchang Medical College, Jiangxi Key Laboratory of Oncology (2024SSY06041), Jiangxi Clinical Research Center for Cancer, Jiangxi Health Commission (JXHC) Key Laboratory of Tumor Microenvironment and Immunoregulation, Jiangxi Key Laboratory of Tumor Metastasis of Jiangxi Health Commission, Nanchang, China; 5 Department of Clinical Pharmacy, Shanghai General Hospital, Shanghai Jiao tong University School of Medicine, Shanghai, China; 6 Department of Radiation Oncology, Shanghai General Hospital, Shanghai Jiao Tong University School of Medicine, Shanghai, China

**Keywords:** axillary pathologic complete response, breast cancer, neoadjuvant therapy, neutrophil-to-lymphocyte ratio, nomogram

## Abstract

**Background:**

Reliable and accessible tools for estimating axillary pathologic complete response (ax-pCR) after neoadjuvant therapy (NAT) remain limited. This study evaluated whether routinely available biomarkers measured before and after NAT provide complementary information for ax-pCR prediction.

**Methods:**

We conducted a retrospective study of patients with clinically node-positive, non-metastatic breast cancer who underwent NAT followed by surgery. Clinicopathological characteristics, treatment information, serum albumin, and inflammatory indices measured before and after NAT were evaluated. Biomarker cutoffs were selected using the maximum Youden index. Variables with P<0.05 in univariable analyses were entered into multivariable logistic regression. A nomogram was constructed from variables that remained significant in the initial multivariable model. Discrimination, calibration, clinical net benefit and optimism-corrected performance were assessed using bootstrap resampling and decision-curve analysis.

**Results:**

Among 158 patients, ax-pCR occurred in 74 (46.8%). In the initial multivariable model, HER2 positivity and pre-NAT albumin ≥43.95 g/L were associated with higher odds of ax-pCR, whereas clinical stage III and post-NAT NLR ≥4.58 were associated with lower odds. The four-variable nomogram achieved an apparent AUC of 0.782 (95% CI, 0.708–0.851). At the maximum-Youden-index threshold, sensitivity was 74.3% (95% CI, 63.3%–82.9%) and specificity was 73.8% (95% CI, 63.5%–82.0%). After bootstrap correction, the AUC was 0.766, the calibration slope was 0.908 and the Brier score was 0.200. Decision-curve analysis suggested potential net benefit across a range of threshold probabilities.

**Conclusion:**

Clinical characteristics and blood-based biomarkers measured before and after NAT provided complementary information for ax-pCR estimation. The nomogram showed moderate discrimination and reasonable internal calibration. External validation is required before clinical application.

## 1 Introduction

Breast cancer is the most commonly diagnosed malignancy among women worldwide (1). For patients with clinically node-positive or locally advanced disease, neoadjuvant therapy (NAT) is an important component of multidisciplinary treatment. NAT can downstage the tumor and axilla, increase opportunities for breast-conserving surgery and provide an *in vivo* assessment of treatment response (2–4). Pathologic complete response (pCR) after NAT is associated with favorable long-term outcomes, particularly in treatment-sensitive breast cancer subtypes (5, 6). Advances in systemic therapy have further increased pCR rates, making response-adapted surgical management increasingly relevant (7, 8).

Improved treatment response has created a particular challenge in managing the axilla. Axillary lymph node dissection (ALND) has traditionally been performed in patients with clinically node-positive disease but may cause lymphoedema, chronic pain and impaired arm mobility (9, 10). Sentinel lymph node biopsy (SLNB) is less invasive, although its accuracy after NAT remains a concern in patients who were initially node-positive (11, 12). Prospective studies, including SENTINA and ACOSOG Z1071, demonstrated that SLNB may be feasible when procedural safeguards such as dual-tracer mapping and targeted node retrieval are applied (11–13). However, these techniques are not universally available. Improved preoperative estimation of axillary pathologic complete response (ax-pCR) may therefore support the selection of patients for less extensive axillary staging.

Several approaches have been investigated for predicting response to NAT, including genomic profiling, circulating tumor DNA and radiomics (14–17). However, their routine application may be limited by cost, technical requirements and availability. Standard clinicopathological characteristics, including hormone receptor status, HER2 status and surrogate molecular subtype, remain the most accessible predictors. Nevertheless, these tumor-related characteristics are largely static and may not fully capture the host response during treatment.

Systemic inflammatory and immune status may provide additional information about treatment response (18). Serum albumin may also reflect the patient’s nutritional and systemic inflammatory condition. Previous studies have reported associations between these biomarkers and overall pCR, but the findings have been inconsistent, and evidence focusing specifically on ax-pCR remains limited (19, 20). Moreover, most studies have evaluated pretreatment measurements alone. Whether pre- and post-NAT biomarkers provide complementary information for ax-pCR prediction therefore remains unclear.

To address this gap, we evaluated inflammatory indices and serum albumin before and after NAT together with clinicopathological characteristics in patients with clinically node-positive breast cancer. We subsequently developed and internally validated a nomogram for estimating ax-pCR probability. The model was intended to support risk stratification and further clinical validation, rather than to serve as a stand-alone basis for omitting axillary surgery.

## 2 Methods

### 2.1 Patients

We retrospectively identified adult women with breast cancer who received initial treatment at Jiangxi Cancer Hospital between January 2014 and December 2022 and completed NAT and subsequent surgery by December 2023.Patients were eligible if they met all of the following criteria (1): age ≥18 years (2); KPS ≥80 (3); pathologically confirmed primary breast cancer (4); clinically node-positive disease (cN1–3) without distant metastasis (M0) before NAT; and (5) receipt of at least two cycles of NAT. Patients were excluded for bilateral breast cancer, another concurrent malignancy, severe organ dysfunction, systemic autoimmune disease, previous systemic anticancer treatment, or incomplete clinical data. The study was approved by the Institutional Review Board of Jiangxi Cancer Hospital (approval no. 2023KY160), which waived the requirement for informed consent.

### 2.2 Data collection and staging

Data collected included age, menopausal status, KPS, tumor characteristics, treatment details and surgical procedures. Clinical TNM stage was assigned according to the eighth edition of the AJCC Cancer Staging Manual based on pretreatment imaging findings. Peripheral venous blood samples were obtained within one week before NAT initiation and within one week before surgery. Neutrophil, lymphocyte, monocyte and platelet counts, as well as serum albumin concentrations, were recorded at both time points.

### 2.3 Pathology and subtyping

Hormone receptor and HER2 status were assessed by immunohistochemistry on baseline core-biopsy specimens. ER and PR positivity were defined as nuclear staining in ≥1% of tumor cells. HER2 positivity was defined as IHC 3+ or IHC 2+ with gene amplification confirmed by FISH. Ki-67 expression was categorized as <14% or ≥14%.For analysis, HER2-positive tumors were classified as the HER2-positive subtype regardless of HR status. HER2-negative tumors were classified as Luminal A, Luminal B or TNBC according to HR and Ki-67 findings.

### 2.4 Blood-based biomarkers

The neutrophil-to-lymphocyte ratio (NLR), platelet-to-lymphocyte ratio (PLR), lymphocyte-to-monocyte ratio (LMR) and systemic immune-inflammation index (SII) were calculated as follows: NLR = neutrophil count/lymphocyte count; PLR = platelet count/lymphocyte count; LMR = lymphocyte count/monocyte count; and SII = platelet count × neutrophil count/lymphocyte count. Serum albumin was measured directly. Changes in each inflammatory index and serum albumin during NAT were calculated as the post-NAT value minus the pre-NAT value and categorized as increased or decreased. ROC analysis was used to determine study-specific cutoff values for the pre- and post-NAT continuous biomarkers. Cutoffs corresponding to the maximum Youden index (sensitivity + specificity − 1) were selected for subsequent dichotomization.

### 2.5 Treatment and outcome

NAT regimens were selected according to tumor phenotype and multidisciplinary assessment. Anthracycline- and taxane-based chemotherapy was commonly used, and HER2-targeted therapy was recorded as received or not received. After NAT, all patients underwent either breast-conserving surgery or modified radical mastectomy, together with axillary lymph node dissection. The primary endpoint was axillary pathologic complete response (ax-pCR), defined as ypN0 without axillary macrometastasis or micrometastasis.

### 2.6 Statistical analysis

Statistical analyses were performed using Python 3.12.13, with patients treated as the independent units of analysis. Categorical variables were compared using Pearson’s chi-square test or Fisher’s exact test, as appropriate. Variables with P<0.05 in univariable analyses were entered into a multivariable logistic regression model; molecular subtype was excluded because of its collinearity with HER2 status. A four-variable nomogram was constructed from variables that remained significant in the initial multivariable model. Model performance was assessed using the AUC, calibration slope, calibration intercept, Brier score and decision-curve analysis, with optimism correction based on 1,000 bootstrap resamples. All tests were two-sided, and P<0.05 was considered statistically significant.

## 3 Results

### 3.1 Patient characteristics

A total of 158 patients with clinically node-positive, non-metastatic breast cancer were included in the analysis. The median age was 49.5 years (range, 22–74 years), and 84 patients (53.2%) were premenopausal. Most patients had stage III disease (125, 79.1%), including 71 (44.9%) with cN2 disease. Invasive carcinoma accounted for 156 cases (98.7%). ER, PR and HER2 positivity were observed in 86 (54.4%), 76 (48.1%) and 63 (39.9%) patients, respectively, while 148 patients (93.7%) had Ki-67 expression of ≥14%. Histological grade was unavailable for 97 patients (61.4%). The molecular subtype distribution included 63 HER2-positive tumors (39.9%), 6 Luminal A tumors (3.8%), 56 Luminal B tumors (35.4%) and 33 triple-negative tumors (20.9%).Regarding treatment, 110 patients (69.6%) received chemotherapy alone, whereas 48 (30.4%) had recorded exposure to targeted therapy. Twelve patients (7.6%) received fewer than four chemotherapy cycles, and 146 (92.4%) received four or more cycles. Modified radical mastectomy was performed in 143 patients (90.5%), while 15 patients (9.5%) underwent breast-conserving surgery, including 8 (5.1%) who received intraoperative radiotherapy. The baseline demographic, clinicopathological and treatment characteristics of the cohort are summarized in **Table 1**.

**Table 1:** Baseline characteristics of the patients (N = 158).

Characteristics	n (%)/summary
Age (years)	Mean ± SD: 48.8 ± 10.2
	Median (range): 49.5 (22–74)
Age group	
<49.5	79 (50.0%)
>=49.5	79 (50.0%)
Tumor laterality	
Left	94 (59.5%)
Right	64 (40.5%)
Menopausal status	
Premenopausal	84 (53.2%)
Postmenopausal	74 (46.8%)
Clinical T stage	
cT1-2	112 (70.9%)
cT3-4	46 (29.1%)
Clinical N stage	
cN1	60 (38.0%)
cN2	71 (44.9%)
cN3	27 (17.1%)
Clinical TNM stage	
Stage II	33 (20.9%)
Stage III	125 (79.1%)
Histological type	
Invasive carcinoma	156 (98.7%)
Ductal carcinoma in situ	2 (1.3%)
Histological grade	
Grade 2	46 (29.1%)
Grade 3	15 (9.5%)
Unknown	97 (61.4%)
ER status	
Positive	86 (54.4%)
Negative	72 (45.6%)
PR status	
Positive	76 (48.1%)
Negative	82 (51.9%)
HER2 status	
Positive	63 (39.9%)
Negative	95 (60.1%)
Ki-67 expression	
<14%	10 (6.3%)
>=14%	148 (93.7%)
Molecular subtype	
HER2-positive	63 (39.9%)
Luminal A	6 (3.8%)
Luminal B	56 (35.4%)
TNBC	33 (20.9%)
Neoadjuvant therapy	
Chemotherapy alone	110 (69.6%)
Chemotherapy + targeted therapy (recorded)	48 (30.4%)
Chemotherapy cycles	
<4	12 (7.6%)
>=4	146 (92.4%)
Surgical approach	
Modified radical mastectomy	143 (90.5%)
Breast-conserving surgery + ALND	7 (4.4%)
Breast-conserving surgery + ALND + IORT	8 (5.1%)

SD, standard deviation; ER, estrogen receptor; PR, progesterone receptor; HER2, human epidermal growth factor receptor 2; TNBC, triple-negative breast cancer; ALND, axillary lymph node dissection; BCS, breast-conserving surgery; IORT, intraoperative radiotherapy.

### 3.2 Factors associated with ax-pCR

**Figure 1:**
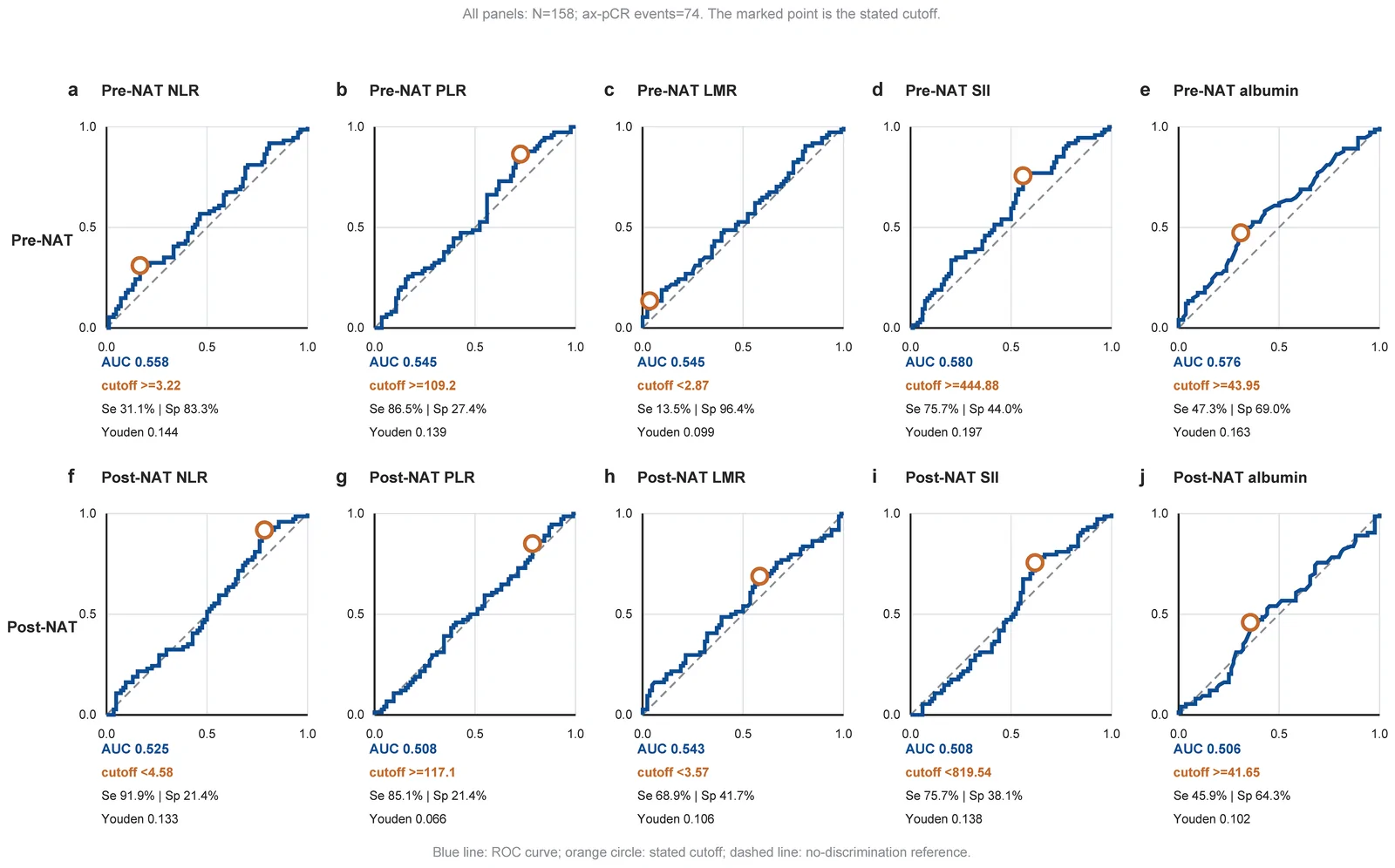
ROC curves and Youden index-based selection of optimal cutoff values for pre- and post-NAT inflammatory hematological indices. Panels **(a–e)** show the ROC curves for pre-NAT NLR, PLR, LMR, SII, and albumin, respectively; panels **(f–j)** show the corresponding post-NAT indices. Orange circles indicate the optimal cohort-specific cutoff values that maximized the Youden index (sensitivity + specificity − 1) in the derivation cohort. Each panel reports the AUC, sensitivity, specificity, and Youden index at the optimal cutoff.

ROC analysis was performed to determine the optimal thresholds for the inflammatory hematological indices. For each biomarker, the optimal cutoff was defined as the value that maximized the Youden index (sensitivity + specificity − 1) in the derivation cohort. The resulting pre- and post-NAT cutoff values were 3.22 and 4.58 for NLR, 109.20 and 117.10 for PLR, 2.87 and 3.57 for LMR, 444.88 and 819.54 for SII, and 43.95 and 41.65 g/L for albumin, respectively (**Figure 1**). Continuous biomarkers were subsequently dichotomized using these optimal cohort-specific thresholds.

Comparisons between the ax-pCR and non-ax-pCR groups are summarized in **Table 2**. Significant associations with ax-pCR were observed for HER2 status, molecular subtype, clinical TNM stage, targeted therapy, chemotherapy cycles, pre-NAT NLR, PLR, LMR, SII and albumin, and post-NAT NLR (all P<0.05). In contrast, age, menopausal status, ER and PR status, Ki-67 expression, clinical T and N stages, the remaining post-NAT indices, and changes in the hematological indices were not significantly associated with ax-pCR.

**Table 2:** Association of clinicopathological features and inflammatory hematological indices with ax-pCR.

Characteristics	Total (N = 158)	ax-pCR (n=74)	Non-ax-pCR (n=84)	P value
Age (years)				0.750
<49.5	79 (50.0%)	36 (48.6%)	43 (51.2%)	
>=49.5	79 (50.0%)	38 (51.4%)	41 (48.8%)	
Menopausal status				0.833
Premenopausal	84 (53.2%)	40 (54.1%)	44 (52.4%)	
Postmenopausal	74 (46.8%)	34 (45.9%)	40 (47.6%)	
ER status				0.294
Negative	72 (45.6%)	37 (50.0%)	35 (41.7%)	
Positive	86 (54.4%)	37 (50.0%)	49 (58.3%)	
PR status				0.143
Negative	82 (51.9%)	43 (58.1%)	39 (46.4%)	
Positive	76 (48.1%)	31 (41.9%)	45 (53.6%)	
HER2 status				**< 0.001**
Negative	95 (60.1%)	32 (43.2%)	63 (75.0%)	
Positive	63 (39.9%)	42 (56.8%)	21 (25.0%)	
Ki-67 expression				0.751
<14%	10 (6.3%)	4 (5.4%)	6 (7.1%)	
>=14%	148 (93.7%)	70 (94.6%)	78 (92.9%)	
Molecular subtype				**< 0.001**
HER2-positive	63 (39.9%)	42 (56.8%)	21 (25.0%)	
Luminal	62 (39.2%)	23 (31.1%)	39 (46.4%)	
TNBC	33 (20.9%)	9 (12.2%)	24 (28.6%)	
Clinical T stage				0.111
cT1-2	112 (70.9%)	57 (77.0%)	55 (65.5%)	
cT3-4	46 (29.1%)	17 (23.0%)	29 (34.5%)	
Clinical N stage				0.448
cN1	60 (38.0%)	31 (41.9%)	29 (34.5%)	
cN2	71 (44.9%)	33 (44.6%)	38 (45.2%)	
cN3	27 (17.1%)	10 (13.5%)	17 (20.2%)	
Clinical TNM stage				**0.003**
Stage II	33 (20.9%)	23 (31.1%)	10 (11.9%)	
Stage III	125 (79.1%)	51 (68.9%)	74 (88.1%)	
Targeted therapy				**< 0.001**
No	110 (69.6%)	37 (50.0%)	73 (86.9%)	
Yes	48 (30.4%)	37 (50.0%)	11 (13.1%)	
Chemotherapy cycles				**0.029**
<4	12 (7.6%)	2 (2.7%)	10 (11.9%)	
>=4	146 (92.4%)	72 (97.3%)	74 (88.1%)	
Pre-NAT NLR				**0.033**
<3.22	121 (76.6%)	51 (68.9%)	70 (83.3%)	
>=3.22	37 (23.4%)	23 (31.1%)	14 (16.7%)	
Pre-NAT PLR				**0.032**
<109.20	33 (20.9%)	10 (13.5%)	23 (27.4%)	
>=109.20	125 (79.1%)	64 (86.5%)	61 (72.6%)	
Pre-NAT LMR				**0.023**
<2.87	13 (8.2%)	10 (13.5%)	3 (3.6%)	
>=2.87	145 (91.8%)	64 (86.5%)	81 (96.4%)	
Pre-NAT SII				**0.009**
<444.88	55 (34.8%)	18 (24.3%)	37 (44.0%)	
>=444.88	103 (65.2%)	56 (75.7%)	47 (56.0%)	
Pre-NAT albumin (g/L)				**0.035**
<43.95	97 (61.4%)	39 (52.7%)	58 (69.0%)	
>=43.95	61 (38.6%)	35 (47.3%)	26 (31.0%)	
Post-NAT NLR				**0.020**
<4.58	134 (84.8%)	68 (91.9%)	66 (78.6%)	
>=4.58	24 (15.2%)	6 (8.1%)	18 (21.4%)	
Post-NAT PLR				0.288
<117.10	29 (18.4%)	11 (14.9%)	18 (21.4%)	
>=117.10	129 (81.6%)	63 (85.1%)	66 (78.6%)	
Post-NAT LMR				0.168
<3.57	100 (63.3%)	51 (68.9%)	49 (58.3%)	
>=3.57	58 (36.7%)	23 (31.1%)	35 (41.7%)	
Post-NAT SII				0.063
<819.54	108 (68.4%)	56 (75.7%)	52 (61.9%)	
>=819.54	50 (31.6%)	18 (24.3%)	32 (38.1%)	
Post-NAT albumin (g/L)				0.191
<41.65	94 (59.5%)	40 (54.1%)	54 (64.3%)	
>=41.65	64 (40.5%)	34 (45.9%)	30 (35.7%)	
NLR change				0.108
Decreased	60 (38.0%)	33 (44.6%)	27 (32.1%)	
Increased	98 (62.0%)	41 (55.4%)	57 (67.9%)	
PLR change				0.383
Decreased	48 (30.4%)	25 (33.8%)	23 (27.4%)	
Increased	110 (69.6%)	49 (66.2%)	61 (72.6%)	
LMR change				0.566
Decreased	131 (82.9%)	60 (81.1%)	71 (84.5%)	
Increased	27 (17.1%)	14 (18.9%)	13 (15.5%)	
SII change				0.209
Decreased	77 (48.7%)	40 (54.1%)	37 (44.0%)	
Increased	81 (51.3%)	34 (45.9%)	47 (56.0%)	
Albumin change				0.857
Decreased	110 (69.6%)	51 (68.9%)	59 (70.2%)	
Increased	48 (30.4%)	23 (31.1%)	25 (29.8%)	

ax-pCR, axillary pathologic complete response; ER, estrogen receptor; PR, progesterone receptor; HER2, human epidermal growth factor receptor 2; TNBC, triple-negative breast cancer; NAT, neoadjuvant therapy; NLR, neutrophil-to-lymphocyte ratio; PLR, platelet-to-lymphocyte ratio; LMR, lymphocyte-to-monocyte ratio; SII, systemic immune-inflammation index. Pearson chi-square tests were used unless an expected cell count was <5, in which case Fisher’s exact test was used. P values are two-sided.

Bold values indicate statistical significance (P < 0.05).

Variables with a univariable P value <0.05 were entered simultaneously into the multivariable logistic regression model. Molecular subtype was excluded because of its structural collinearity with HER2 status. The univariable and multivariable regression results are presented in **Table 3**. In the multivariable analysis, HER2 positivity (adjusted OR, 4.801; 95% CI, 1.250–18.438; P = 0.022) and pre-NAT albumin ≥43.95 g/L (adjusted OR, 2.731; 95% CI, 1.160–6.429; P = 0.021) were independently associated with higher odds of ax-pCR. Conversely, clinical stage III disease (adjusted OR, 0.213; 95% CI, 0.078–0.585; P = 0.003) and post-NAT NLR ≥4.58 (adjusted OR, 0.111; 95% CI, 0.028–0.447; P = 0.002) were independently associated with lower odds of ax-pCR.

**Table 3:** Univariable and multivariable logistic regression analyses of factors associated with ax-pCR.

Characteristics	Univariable OR	95% CI	P value	Multivariable OR	95% CI	P value
HER2 positive	3.938	2.005-7.732	**<0.001**	4.801	1.250-18.438	**0.022**
Targeted therapy received	6.636	3.040-14.487	**<0.001**	1.558	0.381-6.381	0.537
Clinical stage III	0.300	0.131-0.683	**0.004**	0.213	0.078-0.585	**0.003**
Chemotherapy cycles >=4	4.865	1.030-22.978	**0.046**	5.291	0.864-32.412	0.072
Pre-NAT NLR >=3.22	2.255	1.059-4.803	**0.035**	1.983	0.659-5.968	0.223
Pre-NAT PLR >=109.20	2.413	1.062-5.485	**0.035**	1.742	0.569-5.339	0.331
Pre-NAT LMR >=2.87	0.237	0.063-0.897	**0.034**	0.276	0.043-1.749	0.172
Pre-NAT SII >=444.88	2.449	1.236-4.853	**0.010**	1.550	0.580-4.143	0.382
Pre-NAT albumin >=43.95	2.002	1.045-3.834	**0.036**	2.731	1.160-6.429	**0.021**
Post-NAT NLR >=4.58	0.324	0.121-0.866	**0.025**	0.111	0.028-0.447	**0.002**
Molecular subtype: Luminal vs HER2-positive	0.295	0.141-0.615	**0.001**	Excluded	Collinear with HER2	NA
Molecular subtype: TNBC vs HER2-positive	0.188	0.074-0.474	**<0.001**	Excluded	Collinear with HER2	NA

ax-pCR, axillary pathologic complete response; OR, odds ratio; CI, confidence interval; HER2, human epidermal growth factor receptor 2; TNBC, triple-negative breast cancer; NAT, neoadjuvant therapy; NLR, neutrophil-to-lymphocyte ratio; PLR, platelet-to-lymphocyte ratio; SII, systemic immune-inflammation index. Bold values indicate statistical significance (P < 0.05).

### 3.3 Nomogram development and performance

**Figure 2:**
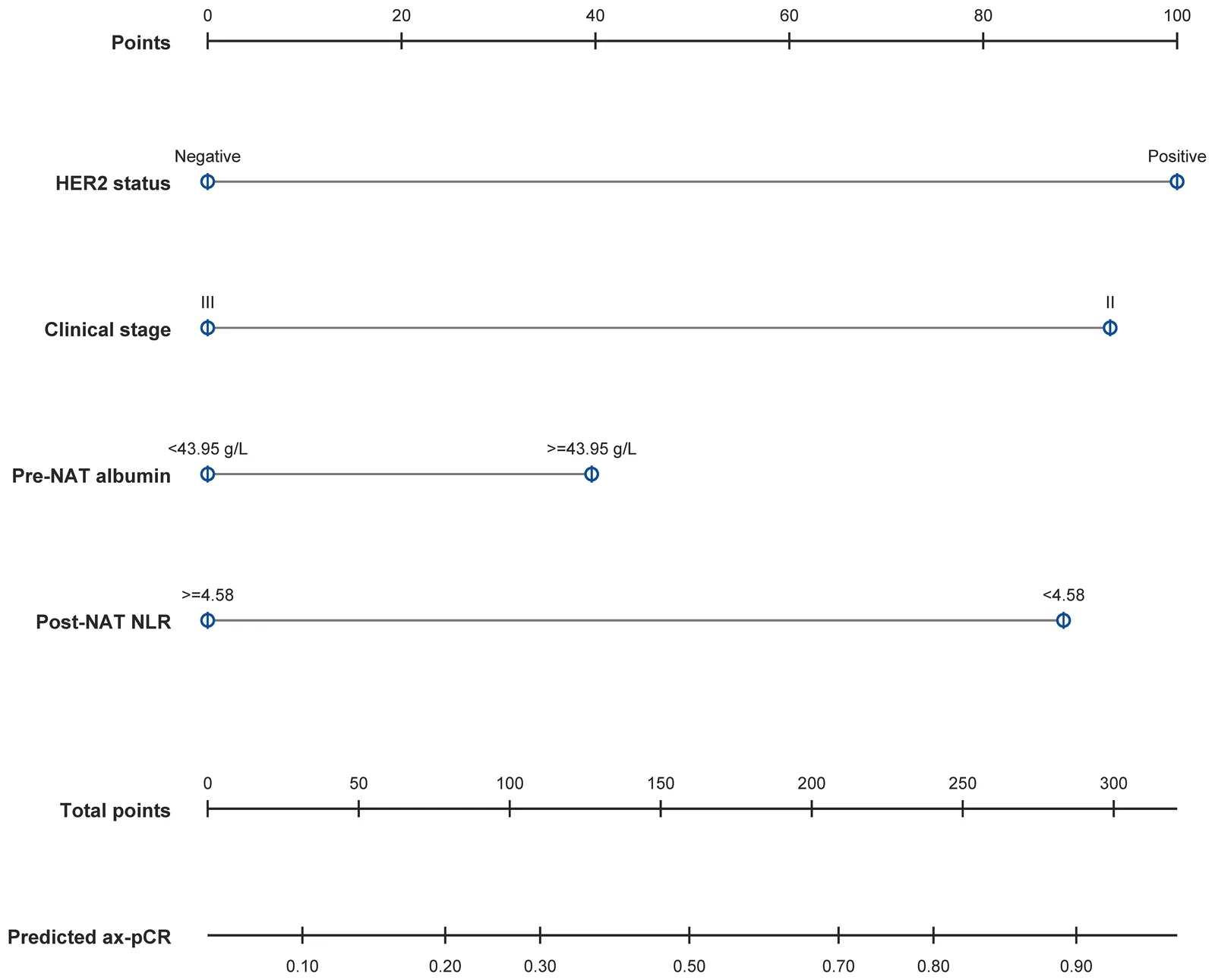
Nomogram for axillary pathologic complete response. The nomogram estimates the probability of ax-pCR after NAT using HER2 status, clinical stage, pre-NAT albumin, and post-NAT NLR. Points assigned to each predictor are summed and mapped to the predicted probability.

Based on the four variables that were statistically significant in the initial multivariable model, a reduced logistic regression model was refitted and presented as a nomogram (**Figure 2**). The nomogram incorporated HER2 status, clinical TNM stage, pre-NAT albumin, and post-NAT NLR to estimate the individual probability of ax-pCR. Although pre-NAT albumin became borderline in the reduced model (OR, 1.973; 95% CI, 0.939–4.144; P = 0.073), it was retained according to the model-construction procedure.

**Figure 3:**
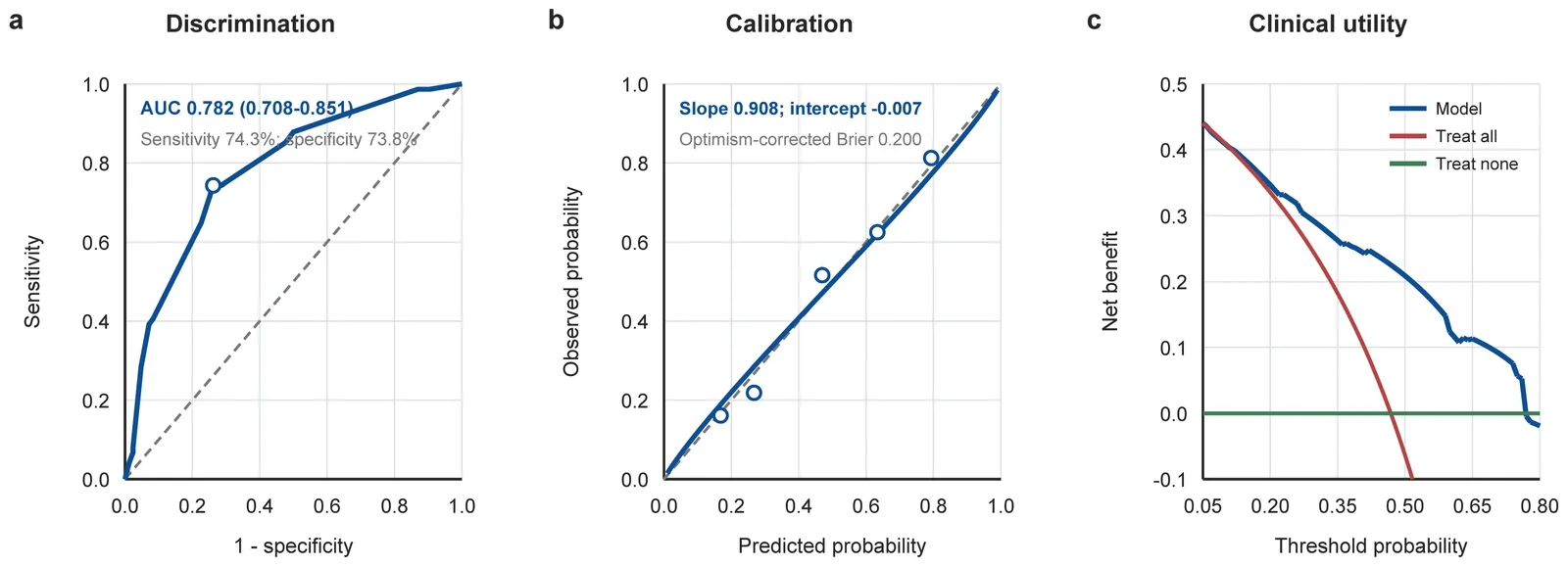
Internal performance of the four-variable ax-pCR model. **(A)** Receiver operating characteristic curve in 158 patients. The apparent AUC was 0.782 (95% CI 0.708-0.851). **(B)** Calibration plot showing observed proportions within fifths of predicted risk and the bootstrap-corrected calibration relationship. The corrected slope was 0.908, the intercept was -0.007, and the Brier score was 0.200. **(C)** Decision-curve analysis comparing the model with treat-all and treat-none strategies from threshold probabilities of 0.05 to 0.80. Decision-curve results are exploratory and require external validation.

The reduced model showed moderate apparent discrimination, with an AUC of 0.782 (95% CI, 0.708–0.851; **Figure 3A**). At the maximum-Youden-index threshold, the sensitivity was 74.3% (95% CI, 63.3%–82.9%) and the specificity was 73.8% (95% CI, 63.5%–82.0%). After 1,000 bootstrap resamples, the optimism-corrected AUC was 0.766.The optimism-corrected calibration slope was 0.908, with an intercept of −0.007 and a Brier score of 0.200 (**Figure 3B**). Decision-curve analysis suggested a potential net benefit of the model compared with the treat-all and treat-none strategies across a range of threshold probabilities (**Figure 3C**). These findings represent internal model performance and require external validation before clinical application.

## 4 Discussion

This study evaluated clinicopathological characteristics and routinely available blood biomarkers for estimating ax-pCR after NAT in clinically node-positive breast cancer. Ax-pCR occurred in 46.8% of patients, with the highest rate observed in HER2-positive disease. HER2 status, clinical stage, pre-NAT albumin and post-NAT NLR remained significant in the initial multivariable model. These findings indicate that clinical characteristics and biomarkers measured at different treatment time points may provide complementary information for axillary response assessment.

HER2 positivity was associated with higher odds of ax-pCR, consistent with previous studies identifying HER2 status as an important determinant of nodal response (21–24). This association may reflect both the biological sensitivity of HER2-positive tumors and the effectiveness of HER2-targeted therapy. Targeted therapy was associated with ax-pCR before adjustment but was not independently significant in the full model. This result should not be interpreted as evidence of limited treatment benefit because receipt of targeted therapy was strongly linked to HER2 status, and treatment selection was not randomized. The small size of some molecular subtype groups and heterogeneity in HER2-targeted treatment limited further subtype-specific and treatment-specific analyses. Clinical stage III disease was associated with lower odds of ax-pCR than stage II disease. This finding is consistent with reports linking greater baseline locoregional disease burden to lower rates of nodal clearance after NAT (25, 26). Thus, clinical stage may complement tumor biology by reflecting the pretreatment tumor and nodal burden. Nevertheless, this association was derived from a single-center cohort and requires confirmation in broader patient populations. Pre-NAT albumin ≥43.95 g/L was associated with higher odds of ax-pCR in the initial multivariable model. Serum albumin may reflect nutritional condition, systemic inflammation and the ability to tolerate planned systemic treatment (27). However, albumin became borderline after the reduced four-variable model was refitted. This change indicates sensitivity to model specification and suggests that albumin should be regarded as a candidate contributor rather than a firmly established independent predictor.

The association between NLR and ax-pCR differed according to the measurement time point. Pre-NAT NLR was associated with ax-pCR in the univariable analysis, but this association did not remain significant after multivariable adjustment. Previous studies have also reported inconsistent associations between baseline NLR and response to NAT (28–30). Differences in molecular subtype, treatment regimen, sample size and cutoff selection may contribute to this heterogeneity. In contrast, elevated post-NAT NLR was independently associated with lower odds of ax-pCR. This association may reflect residual systemic inflammation or treatment-related changes in peripheral blood neutrophil and lymphocyte counts (31–34). However, the observational design does not establish a biological mechanism, and the change in NLR itself was not significantly associated with ax-pCR. Pre-NAT NLR may reflect the baseline systemic inflammatory state, whereas post-NAT NLR may capture inflammatory and hematologic changes after treatment.

The four-variable nomogram showed moderate apparent discrimination and reasonable bootstrap-corrected calibration. Decision-curve analysis also suggested potential net benefit across a range of threshold probabilities. Previous ax-pCR models have incorporated molecular subtype, disease burden, breast response and other clinicopathological variables (21–26). The potential value of the present model lies in combining routinely available clinical information with pre- and post-NAT blood biomarkers at minimal additional cost. However, no direct comparison with published models was performed. Superiority over existing models and generalizability to other clinical settings therefore cannot be inferred. Clinically, the nomogram may provide an accessible framework for preoperative risk stratification and patient counseling. It could also help identify patient groups for future prospective studies of less extensive axillary management. Nevertheless, the model should not be used as a stand-alone basis for omitting ALND. The decision-curve analysis was performed in the development cohort, and the clinical consequences of false-negative predictions may vary across surgical strategies and institutions.

Several limitations should be acknowledged. The retrospective single-center design and modest sample size increase the risks of selection bias, residual confounding, and overfitting. Histological grade was unavailable for 61.4% of patients, and treatment regimens and targeted therapy exposure were heterogeneous. Biomarker cutoffs were derived in the same cohort and not reselected during bootstrap resampling, while dichotomization may have caused information loss and additional optimism. Interactions and adequately powered subgroup analyses were not performed, and external validation and comparisons with existing models are lacking. Independent multicenter validation and prospective evaluation are therefore needed to establish the model’s clinical utility and oncological safety.

## 5 Conclusion

In this retrospective cohort of clinically node-positive breast cancer, HER2 status, clinical stage, pre-NAT albumin and post-NAT NLR were selected from the initial multivariable model to develop a nomogram for ax-pCR estimation. The model showed moderate discrimination, reasonable bootstrap-corrected calibration and potential exploratory clinical net benefit. This nomogram may provide an accessible framework for preoperative ax-pCR risk stratification, but external validation and prospective evaluation are required before it can inform axillary surgical de-escalation or omission of ALND.

## Data Availability

The original contributions presented in the study are included in the article/supplementary material. Further inquiries can be directed to the corresponding authors.
